# Diversity and effect of *Trichoderma* spp. associated with green mold disease on *Lentinula edodes* in China

**DOI:** 10.1002/mbo3.364

**Published:** 2016-05-04

**Authors:** Gangzheng Wang, Xiantao Cao, Xiaolong Ma, Mengpei Guo, Changhao Liu, Lianlian Yan, Yinbing Bian

**Affiliations:** ^1^ Institute of Applied MycologyHuazhong Agricultural UniversityNo. 1 Shizishan RdWuhanHubei430070China; ^2^ Key Laboratory of Agro‐Microbial Resource and DevelopmentMinistry of AgricultureWuhanChina

**Keywords:** Effect of *Trichoderma* spp. on *L. edodes*, green mold, *Lentinula edodes*, species diversity and distribution, *Trichoderma* spp.

## Abstract

*Lentinula edodes*, one of the most important edible mushrooms in China, is affected heavily by the infection of green mold that overgrows mushroom mycelia. We collected the diseased samples from main *L. edodes* cultivation regions in China to characterize the pathogen and to study the effect of *Trichoderma* spp. on *L. edodes* species. We identified six *Trichoderma* species, that is, *T. harzianum*,* T. atroviride*,* T. viride*,* T. pleuroticola*,* T. longibrachiatum,* and *T. oblongisporum* based on the internal transcribed spacer or tef1‐*α* sequences and morphology characteristics. In confrontation cultures on Petri plates or in tubes, and in *L. edodes* cultures in a medium containing *Trichoderma* metabolites, *L. edodes* mycelia were not only distorted and swollen, but also inhibited by *Trichoderma* isolates. It is not possible that adjusting pH value or temperature is used for controlling *L. edodes* green disease, because the growth of most of *Trichoderma* isolates and *L. edodes* shared similar pH and temperature conditions.

## Introduction

The fact that *Trichoderma* spp. can affect *Agaricus bisporus* was first described by Sinden and Hauser (1953). The disease did not receive much attention first primarily due to its infection in small areas of mushroom beds. However, a new severe problem was detected in numerous mushroom farms in Northern Ireland in 1985 where *Trichoderma aggressivum* causes green mold disease in *A. bisporus* (Seaby 1987). It appeared later in North America in 1992 that, first described and (Rinker et al. 1997). *T*. *aggressivum* rapidly overgrows compost and *A. bisporus* mycelium, and generates a wealth of green conidia to inhibit the formation of *A. bisporus* fruiting bodies, leading to a large reduction in mushroom yield (Anderson et al. 2001).

From the perspective of *L. edodes*, green mold induced by *Trichoderma* can attack and kill *L. edodes* mycelia in the bed‐logs and reduce the mushroom yield. The main species detected in damaged bedlogs were *T. harzianum* Rifai and *T. polysporum* Rifai (Komatsu 1976). Subsequently, a number of researchers explored environmental factors and cultivation conditions, for instance, carbon and nitrogen source or temperature and water, for their effect on mycelial growth of *L. edodes* and *Trichoderma* spp. (Tokimoto and Komatsu 1979; Badham 1991). Simultaneously, Bruce et al. (1984) documented that a volatile of *Trichoderma* spp. could inhibit *L. edodes* mycelial growth. Selection and breeding studies of *L. edodes* species resistant to *Trichoderma* spp. have been conducted by many researchers (Tokimoto. et al. 1984; Lee Hye‐Min et al. 2008). Nevertheless, the studies of the effect of *Trichoderma* species on *L. edodes* are less well‐documented. To the best of our knowledge, the main *Trichoderma* species affecting *L. edodes* are *T. harzianum*,* T. viride*,* T. longibrachiatum,* and *T. polysporum* in Fujian province (Jiang et al. 1995). *L. edodes*, the second most important edible mushroom in worldwide production, is widely cultivated in China. Therefore, it is important to collect and identify *Trichoderma* spp. in the main cultivation areas.

In this study, we collected a wealth of green mold disease logs from main Shiitake cultivation areas in China. Based on the morphology and internal transcribed spacer (ITS) sequence as well as tef1‐*α* sequence, we analyzed the species and distribution of *Trichoderma* spp. Additionally, we undertook the mycelial growth under different conditions and effect of *Trichoderma* spp. on *L. edodes* via scanning electron microscope. On the basis of this study, we will identify the best *L. edodes* strain to elaborate the mechanism of the interaction between *L. edodes* and *Trichoderma* spp. by the transcriptomic analysis.

## Materials and Methods

### Collection of green mold disease logs and isolation of fungal species

Between 2012 and 2013, green mold disease occurred continuously in *L. edodes* production areas in Suizhou, Hubei, leading to a significant negative effect on the development of *L. edodes*. Fifty‐nine *Trichoderma* isolates were collected from the diseased logs of *L. edodes* in four main cultivation areas (Table 1), and deposited in the culture collection of the Institute of Applied Mycology, Huazhong Agricultural University. All species were isolated and maintained according to Hatvani et al. (2007).

**Table 1:** The numbers and origin of *Trichoderma* spp. isolates used in the study

Origin	Strains						Sum
	*T. harzianum*	*T. viride*	*T. atroviride*	*T. longibrachiatum*	*T. pleuroticola*	*T. oblongisporum*	
Suizhou,Hubei	T8,T11,T12,T14,T15,T18,T19,T27,T32,T36,T44,T62		T25,T40,T59	T33,T43,T47		T37,T60,T61	21
Wuhan,Hubei	T5,T48						2
Shiyan,Hubei	T38,T46,T49						3
Biyang,Henan	T7,T9,T16	T13,T23	T29,T30				7
Xixia,Henan	T31,T50,T51						3
Sanmenxia,Henan	T53,T54,T55	T52,T56		T57			6
Lishui,Zhejiang	T20,T21,T26,T42						4
Qingyuan,Zhejiang	T2				T35,T39		3
Jingning,Zhejiang	T4,T28,T41						3
Ningde,Fujian	T1,T10		T24				3
Youxi,Fujian	T3						1
Minqing,Fujian	T6,T17				T22		3
Sum	39	4	6	4	3	3	59

### Species identification

#### Morphology analysis

The isolated species were incubated on complete yeast medium (CYM; 2% glucose, 0.2% yeast extract, 0.2% peptone, 0.046% KH_2_PO_4_, 0.1% K_2_HPO_4_, 0.05% MgSO4·7H_2_O) at 25°C in darkness, during which colony shape and pigment were documented. Simultaneously, four cover glasses were inserted slantingly into the CYM medium to observe the conidia and conidiophores via the microscope (U‐RFL‐T, Olympus) when the mycelia spread on the cover glass (Park et al. 2006).

#### Molecular analysis

Mycelium that has grown in CYM medium was used for DNA isolation using the CTAB (hexadecyltrimethylammonium bromide) method (Zhang et al. 2010). The PCR primer pairs (Table 2) and amplification procedures were used according to the method of Sadfi‐Zouaoui (Sadfi‐Zouaoui et al. 2009). PCR products were tested and sequenced by WuHan tsingke BioTech Co., Ltd. The sequences were inspected and refined manually, and blasted in TrichoBLAST databank. Additionally, the phylogenetic trees of the ITS and tef1‐*α* sequences were constructed with maximum likelihood method by MEGA 6.0.

**Table 2:** Primer species used in this study**.**

Primers	Sequence
ITS‐1	TCCGTAGGTGAACCTGCGG
ITS‐4	TCCTCCGCTTATTGATGC
EF1‐728F	CATCGAGAAGTTCGAGAAGG
EF1‐728R	GCCATCCTTGGAGACCAGC

### Culture characteristics of *Trichoderma* spp. and *L. edodes* species

#### Effect of different temperature treatments on mycelial growth of *Trichoderma* spp. and *L. edodes* species

Twenty‐three isolates were selected as the test species including the six species, *T. harzianum*: T3, T6, T10, T12, T20, T21, T27, T28, T38, T42, T55; *T. atroviride*: T25, T29, T30, T24; *T. viride*: T13, T23, T52; *T. pleuroticola*: T22, T35, T39; *T. longibrachiatum*: T57; and *T. oblongisporum*: T37. Two *L. edodes* species, Yuhua‐2 (mainly cultivated in Biyang, Hubei province of China) and Xiang939 (mainly cultivated in Qinyuan county, Zhejiang province of China), were selected as the control species. The 8 mm mycelial plugs in diameter were inoculated in CYM, and cultured separately at 15°C, 20°C, 25°C, 30°C, and 35°C. Each treatment was replicated for three times. Growth rate (mm/d) = (colony diameter‐plug diameter)/2n, where n represents cultivation days.

#### Effect of different pH on mycelial growth of *Trichoderma* spp. and *L. edodes* species

Two *Trichoderma* species on mushroom, namely the dominant species *T. harzianum* (T36) and the recently reported species *T. oblongisporum* (T37), and *L. edodes* Qiu‐7 were selected as the tested strain. The 8 mm plugs in diameter of testing species were inoculated on CYM separately at pH values of 2, 3, 4, 5, 6, 7, 8, 9, 10, and 11, where different pH values were adjusting via adding HCl or NaOH solution filtered via the biofilter after sterilization. The treatment was repeated four times. The diameters were measured after 2 days (for T36 and T37) and 7 days (for Qiu‐7). Growth rate (mm/d) = (colony diameter‐plug diameter)/2n, where *n* represents cultivation days.

### Effect of *Trichoderma* spp. on *L. edoeds* mycelia

#### Effect of *Trichoderma spp*. on *L. edoeds* mycelia in petri plates

Fifteen of the 59 isolates including six species were chosen to test the aggressiveness of the species. The experiments were carried out with three replicates as follows. Mycelial agar plugs (8 mm in diameter) were cut from the growing front of 7‐day‐old colonies of *L. edodes* species (Qiu‐7 mainly cultivated in Wuhan, Hubei province of China) and were inoculated onto CYM at 1 cm from the edge in Petri plates of 9 cm in diameter. Seven days later, mycelial plugs of *Trichoderma* cultures were inoculated in the same way but on the opposite side 1 cm apart from the plate edge. The confrontation conditions, inhibition rate of *Trichoderma* spp. against *L. edodes* mycelial growth, were observed. Then, the changes of *L. edodes* mycelium treated by *T. harzianum*,* T. oblongisporum,* and *T. atroviride* were observed via SEM (scanning electron microscope).

#### Effect of *Trichoderma* fermentation broth on *L. edodes* mycelium

To test *Trichoderma* fermentation broth effect on *L. edodes* mycelia, *T. oblongisporum* T37 plugs were inoculated into the PD (potato dextrose) broth, followed by 1 week culture in darkness at 25°C, 160 rpm. Mycelium cells were removed by filter paper, and the filtrate was treated by either further filtration via 0.22 *μ*m filter paper. Then, filtrates were added to the CYM medium to thirty percent in volume, with 30% sterile water used for control. *L. edodes* Qiu‐7 mycelium plugs (8 mm in diameter) were inoculated on the medium and cultured in darkness at 25°C. Ten days later, the diameters of the colonies were measured to test the inhibition ratio and mycelium growth rate. Then, the changes of *L. edodes* mycelium were observed via SEM.

### Data analysis

Data obtained were analyzed using statistical IBM SPASS20. (SPSS Inc., Chicago, IL, USA) Statistical significance was identified at the 95% confidence level (*P* < 0.05).

## Results

### Sample collection and survey on *L. edodes* rot log disease

**Figure 1:**
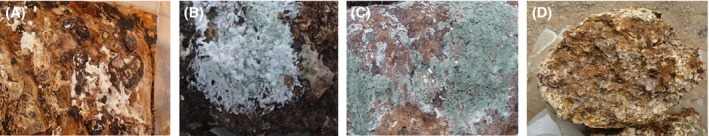
The process of symptom development of *L. edodes* green mold disease.

*L. edodes* logs with green mold disease in Suizhou have the features of sudden outburst and rapid spread, especially after watering in the first flush mushroom. Logs infected by green mold showed similar disease symptoms: bag‐logs infected by the pathogen initially become soft, no green mold appearing, and with *L. edodes* mycelium growing, spots appeared in the bags (Fig. 1A). The spot turned green in the presence of high humidity or with increasing time, produced dark green conidia (Fig. 1B and C). Finally, all of the bag‐logs would become rotten and loose, and the mycelia from the diseased bags produced a mold odor of that pathogen and the good smell of *L. edodes* mycelia disappeared as *L. edodes* mycelia died off (Fig. 1D).

### Morphology identification of the isolates

**Figure 2:**
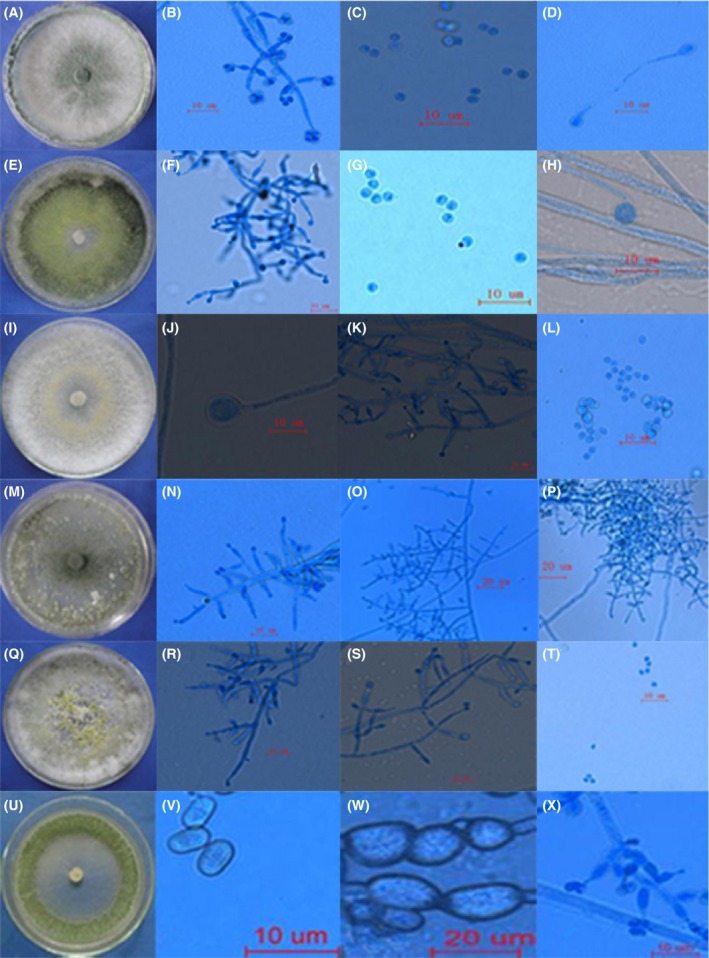
Colony and microscopic characteristics of different *Trichoderma* isolates.

According to the classification method of Bissett (1984), Gams and Bissett (1998) and Park et al. (2006), six *Trichoderma* species, namely *T. harzianum*,* T. atroviride*,* T. viride*,* T. pleuroticola*,* T. longibrachiatum,* and *T. oblongisporum*, were isolated based on the colony shape, conidia, conidiophores size, chlamydospore, and pigment (Fig. 2). First, color of the colony is white, and become light green to dark green later because conidia clusters generating. The mycelial growth speeds of different isolate colony varied from 11.38 mm/d to 17.55 mm/d. Except for *T. pleuroticola* and *T. longibrachiatum*, lots of chlamydospores were found in late stage of the remaining isolate growth. The details of different *Trichoderma* isolate characteristics were seen in the Table 3.

**Table 3:** Colony and microscopic characteristics of different *Trichoderma* isolates

Species	Colony in CYM	Conidiophores and phialides	Conidia	Chlamydospores
*T. harzianum*	11.38 mm/d, powdery, light green, later gray green, floccose, white to grayish (Fig. 2A)	Ampulliform, base constricted, center swollen, peak slender, 1.99–3.43 *μ*m to 3.39–6.87 *μ*m in length (Fig. 2B)	Subglobose to ellipsoidal, smooth‐walled, mostly 2.17–2.83 *μ*m × 2.08–3.87 *μ*m in diameter (Fig. 2C)	Elliptic (Fig. 2D)
*T. viride*	15.17 mm/d, white in the primary stage (Fig. 2E)	Branched irregularly, slightly crooked or hook‐like, base constricted, center swollen, peak slender, 1.47–2.33 *μ*m to 5.66–8.46 *μ*m in length (Fig. 2F)	Spherical or subglobose, 1.79–2.54 *μ*m to 1.91–3.07 *μ*m in diameter (Fig. 2G)	Basidixed and subglobose (Fig. 2H)
*T. atroviride*	17.55 mm/d, light yellow rounded conidial cluster, dark green colony later (Fig. 2I)	4.15–8.33 *μ*m in diameter, and 1.49–2.86 *μ*m in the width of the center, with most single phialides located in the conidiophores (Fig. 2K)	Ellipsoidal to subglobose, and 1.94–3.16 *μ*m to 2.32–3.97 *μ*m in length (Fig. 2L)	Basidixed and subglobose (Fig. 2J)
*T. longibrachiatum*	16.55 mm/d, white at first, septate, and |smooth‐walled, yellow pigment (Fig. 2 M)	Cylindrical, base partly constricted, center slender, and shorter, 4.21–15.67 *μ*m in the length, with the widest part being 1.23–3.95 *μ*m (Fig. 2N, O, P)	Formed in the phialides, green, and ellipsoidal (Fig. 2N, O, P)	Not found
*T. pleuroticola*	15.55 mm/d, white, smooth‐walled, septate, yellow green conidial clusters(Fig. 2Q)	Separately or opposite in the base of the principal axis, longer, base constriction not obvious, 3.88–10.34 *μ*m in length, with the widest segment being 2.20–3.01 *μ*m (Fig. 2R,S)	Subglobose, green, smooth‐walled, 2.20–2.95 *μ*m to 1.88–3.01 *μ*m in diameter (Fig. 2T)	Not found
*T. oblongisporum*	13.5–13.7 mm/d, floccose, white, and septate (Fig. 2U)	Branched at vertical angles, primary branches single or opposite; ampulliform, 3.1–6.7 × 2.7–4.0 *μ*m in length, base constricted, center swollen, and peak slender (Fig. 2X).	Ellipsoidal or oblong, 3.3–4.7 × 2.4–3.2 *μ*m(Fig. 2V)	Subglobose to ellipsoidal (Fig. 2W)

CYM, complete yeast medium.

### Molecular identification of the isolates

The ITS sequence sizes of 59 isolates were 532 bp to 604 bp (Fig. S1), and blasted in TrichoBLAST database. According to the highest similarity, the highest score value and the least e‐value, six *Trichoderma* species, that is *T. harzianum*,* T. atroviride*,* T. viride*,* T. pleuroticola*,* T. longibrachiatum,* and *T. oblongisporum*, were identified. Additionally, the tef1‐*α* sequence was used to identify the recently reported *T. oblongisporum* on mushroom and some *Trichoderma* species not identified via the ITS sequence (Fig. S2).

**Figure 3:**
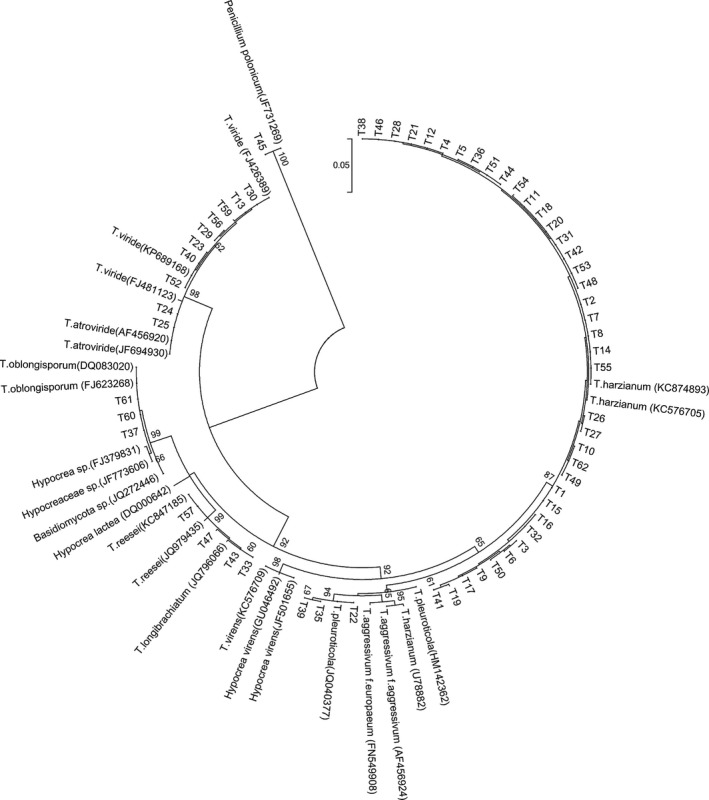
Phylogenetic tree of the 59 *Trichoderma* isolates by maximum likelihood method based on ITS sequences (MEGA 6.0).

**Figure 4:**
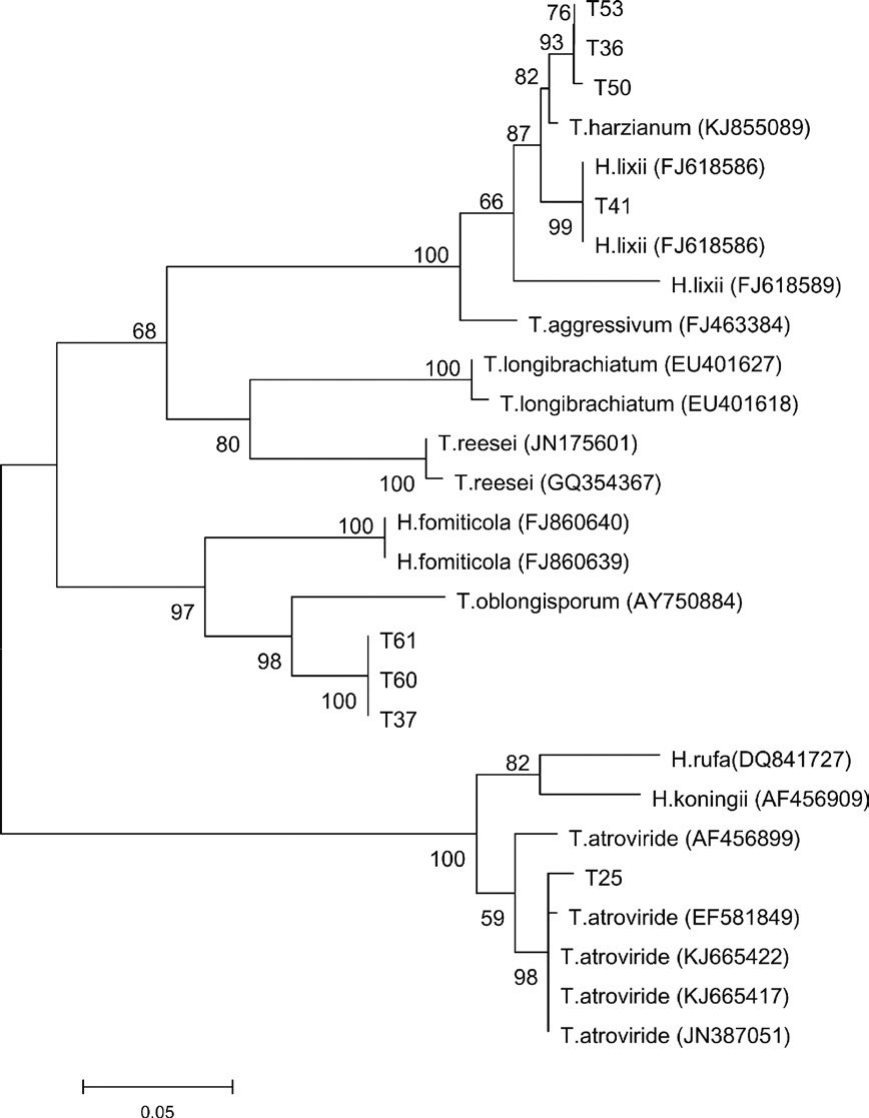
Phylogenetic tree of the 8 *Trichoderma* isolates not identified via ITS sequence by maximum likelihood method based on tef1‐*α* sequences (MEGA 6.0).

The phylogenetic trees of the 59 *Trichoderma* isolates were constructed by maximum likelihood method based on ITS and tef1‐*α* sequences (Figs. 3, 4). The result demonstrated that all species were divided into six groups. T1, T2, T3, T4, T5, T6, T7, T8, T9, T10, T11, T12, T14, T15, T16, T17, T18, T19, T20, T21, T26, T27, T28, T31, T32, T36, T38, T41, T42, T44, T46, T48, T49, T50, T51, T53, T54, T55 , T62 and *T. harzianum* (KC874893, KC576705 and U78882) were classified in the same group. *T. longibrachiatum* (JQ796066) was grouped with the T33, T43, T47 and T57. From the perspective of the ITS sequence, T13, T23, T29, T30, T40, T52, T56 and T59 together with *T. viride* (KP689168, FJ426389) were classified in a group, and T24 and T25 were grouped into *T. atroviride* (AF456920, JF694930) and *T. viride* (FJ481123), while T29, T30, T40 and T59 morphology characteristics showed the higher similarity to those of *T. atroviride*. Therefore, we applied the tef1‐α sequences to identify that species accurately, and the results also suggested that T29, T30, T40 and T59 belonged to the group of *T. atroviride* (EF581849, KJ665422, KJ665417, JN387051). The sequences of T37, T60 and T61 were highly similar to that of *T. oblongisporum* (OQ083020 and FJ623268). T22, T35 and T39 showed the most consistency with *T. pleuroticola* (JQ040377).

### Effect of different temperatures and pH on *L. edodes* and *Trichoderma* spp. mycelia

Of 24 *Trichoderma* species, 18 species could grow at 15–35°C. Furthermore, the time of the sporulation was shorter with temperature increasing. However, several species, such as T52, T37, T42, T29, T30, and T23, stopped growth at 35°C. The growth rate of T39 and T21 peaked at 30°C, whereas that of the remaining species peaked at 25°C; the maximum rate of mycelial growth was 7.05–19.73 mm/d. However, the growth rate of *T. oblongisporum* which has been identified recently in edible fungi was the lowest 7.05 mm/d. From the perspective of *L. edodes* mycelia, Yuhua‐2 and Xiang939 grew well at 25°C, and the average growth rate of mycelia were 5.57 mm/d and 4.16 mm/d, respectively. Neither species grew at 35°C (Fig. S3).

According to Figure S4, we could conclude that the suitable pH of two *Trichoderma* isolates and *L. edodes* Qiu‐7 species was 4–7. However, T36 and T37 could grow normally at a pH range 2–11. What is more, the growth rate of *Trichoderma* isolates was sharply faster than that of *L. edodes* in the same condition, suggesting that it is unlikely to inhibit the growth of *Trichoderma* isolates by adjusting pH values and environment temperature.

### Effect of *Trichoderma spp*. on *L. edodes* mycelia

In dual culture of *L. edodes* and *Trichoderma spp*. on agar plate, differences were observed in the interactions of six *Trichoderma* species with *L. edodes* mycelia when measured by the inhibition rate for *L. edodes* hypha (Fig. S5). *T. harzianum* and *T. pleuroticola* inhibited heavily *L. edodes* mycelium growth, which was verified by the inhibition ratio ranging from 63% to 94%. *T. viride* could inhibit heavily *L. edodes* mycelial growth. Nevertheless, the inhibition effect among different species varied more obviously. The inhibition rates were 73% and 50% for *T. atroviride* isolates T29 and T24, respectively, with the sharpest variations among all species. However, *T. longibrachiatum* manifested inhibition ratio 29%. From the perspective of mycelial morphology, *Trichoderma* mycelia could overgrow and spread on *L. edodes* mycelia, forming irregular conidial clusters, resulting in gradual withering of *L. edodes* mycelia. Besides, various pigment and antagonistic streaks appeared on the back of the medium.

### Changes in *L. edodes* mycelium treated by *Trichoderma* spp. in SEM

**Figure 5:**
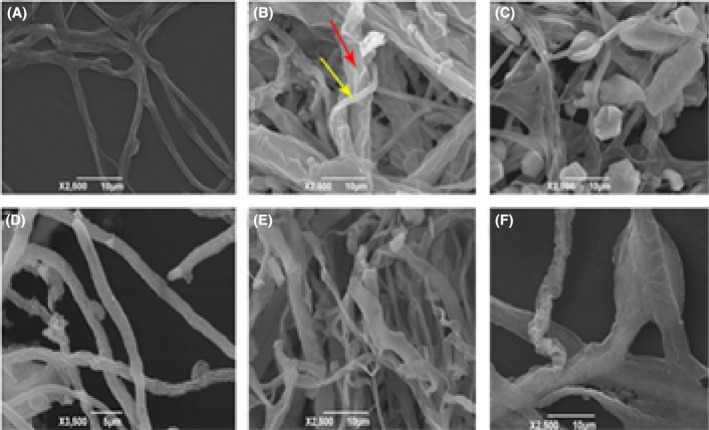
Effect of *Trichoderma* spp. and *T. oblongisporum* metabolites on *L. edodes* mycelia.

From the perspective of the interaction between *Trichoderma* mycelium and *L. edodes* mycelium, we observed that *L. edodes* hyphal cells from the interaction zone are distorted with local swellings, whereas the mycelium untreated by *T*. *harzianum* was smooth and straight (Fig. 5A and B), and *T*. *harzianum* hyphae could coil around the hyphae of *L. edodes* (Fig. 5C) through SEM (scanning electron microscopy) observation. After getting contact to *T. oblongisporum* and *T. atroviride* mycelia, *L. edodes* mycelia became ruptured and rough (Fig. 5D, E) After *L. edodes* mycelium grew about 15d in complete yeast broth medium, the *Trichoderma* plugs were added to the broth. Several days later, the *L. edodes* mycelium balls treated by *Trichoderma* spp. were broken or became smaller. Conversely, the mycelium palls untreated by *Trichoderma* spp. grew normally. Additionally, metabolites of *T. oblongisporum* T37 fermentation broth treated by different methods affected *L. edodes* mycelia growth differently. Compared with the control group, *L. edodes* mycelia that grew in the medium containing 30% *T. oblongisporum* metabolites were dramatically thick (Fig. 5F).

## Discussion

*Lentinula edodes* mycelium in bed‐logs is attacked and killed by *Trichoderma* species, for instance, *T. harzianum* and *T. polysporum,* which produced antifungal substances and mycolytic enzymes that commonly caused injury to mycelial growth and fruit body formation of *L. edodes*, as well as having negative effect on the yield of Shiitake cultivation (Tokimoto 1985; Ulhoa and Peberdy 1992; Seaby 1998). However, little is known about the distribution and species of *Trichoderma* spp. attacking Shiitake mycelium in China. Therefore, we collected many diseased logs from main Shiitake cultivation areas, in order to characterize them and explore the interaction between them.

In this study, colony morphology and conservative sequence including ITS and tef1‐*α* sequence were included to identify those *Trichoderma* species. Six *Trichoderma* species, such as *T. harzianum*,* T. atroviride*,* T. viride*,* T. pleuroticola*,* T. longibrachiatum,* and *T. oblongisporum*, were obtained from the diseased logs in main Shiitake cultivation areas of China. *T. harzianum*, a general and widespread pathogen in mushroom (Tokimoto and Komatsu 1995; Jiang et al. 1995; Savoie 1998; Lee Hye‐Min et al. 2008), accounted for 68% in all *Trichoderma* species isolated from the diseased Shiitake logs, as well as being observed extensively in four cultivation areas. This species is used widely for the biological control of plant pathogen (Steindorff et al. 2014; Troian et al. 2014). *T. atroviride* possessed stronger capacity attacking the *L. edodes* mycelium because of its mycelia overgrowing *L. edodes* mycelia, and was isolated in three cultivation areas except for Zhejiang province. The remaining species were rare in Shiitake logs: the mycelium of Shiitake was attacked weakly by *T. longibrachiatum* in Hubei and Henan; *T. pleuroticola* reported in diseased Oyster mushroom (Park et al. 2006) was only found on Shiitake in Zhejiang and Fujian, demonstrating that it had no host specificity, which may be relative to the environmental factors in two province that are in the subtropics; yet, *T. viride* was only detected on Shiitake in Henan province; simultaneously, *T. oblongisporum* was obtained only in Suizhou, Hubei, and the morphological characteristics and the chlamydospores were sharply different from other *Trichoderma* species (Cao et al. 2014). The environment factor differences, for instance temperature, humidity, sunshine, and the like, were affected sharply by mountains and rivers, and the activity of species varied under different conditions, which may influence the distribution of species. Widden P found that environmental conditions where different *Trichoderma* species live had obvious region distribution and changed as the season varied, which had an important role in *Trichoderma* spp. growth (Widden and Scattolin 1988).*L. edodes* hyphal cells from the interaction zone were thickened and rough with local swellings, which are consistent with the results of the interaction between *Trichoderma* spp. and *L. edodes* (Lee Hye‐Min et al. 2008) and *Sclerotinia sclerotiorum* (Troian et al. 2014). *T*. *oblongisporum* metabolites could get *L. edodes* mycelia thickened, and *L. edodes* hyphal cells got broken in the presence of *Trichoderma* spp., based on which we assumed that *Trichoderma* spp. showed various effects against *L. edodes*: they could inhibit the mycelium growth mainly by coiling or mycoparasitism, or produce a wealth of enzymes and the antibiotics to inhibit *L. edodes* mycelium growth, for instance, chitinase, *β*‐glucanases, anthraquinones, and isocyano metabolites. A battery of hydrolytic enzymes consisting of chitinases, *β*‐glucanases, and proteases produced by different *T*. *aggressivum* could attack mycelial walls of *A. bisporus* (Williams et al. 2003; Guthrie et al. 2005; Guthrie and Castle 2006). The antagonistic role of *Trichoderma* mycelia on those of *L. edodes* were relative to fungal cell wall lytic enzyme activities and ether‐soluble neutral antifungal compounds produced by *Trichoderma* spp. (Ishikawa et al. 1980; Tokimoto 1982), which was consistent with our result that *L. edodes* mycelia were degraded by *Trichoderma* metabolites through scanning electron microscopy observation. To better understand their interaction, we would explore how *L. edodes* is affected at both transcription and protein level via transcriptome and proteome analysis.

## Conflict of Interest

None declared.

## Supporting information


**Figure S1.** The electrophoresis profile of ITS of the isolates.
**Figure S2.** The electrophoresis of tef1‐α of the isolates.
**Figure S3.** Effect of different temperature treatment of mycelia growth of 2 *L. edodes* and *Trichoderma* spp. isolates.
**Figure S4.** Effect of different pH treatment on mycelia growth of two *Trichoderma*isolates and one *L. edodes* strain.
**Figure S5.** Inhibition rate of 6 *Trichoderma*species on *L. edodes* Qiu‐7 in confrontation culture.
