# Supplementary material for: Diversity and effect of Trichoderma spp. associated with green mold disease on Lentinula edodes in China
Source: Microbiologyopen. 2016 May 4;5(4):709–18. doi: 10.1002/mbo3.364 (PMC4985603; doi:10.1002/mbo3.364)

Fig.S1 The electrophoresis profile of ITS of the isolates.

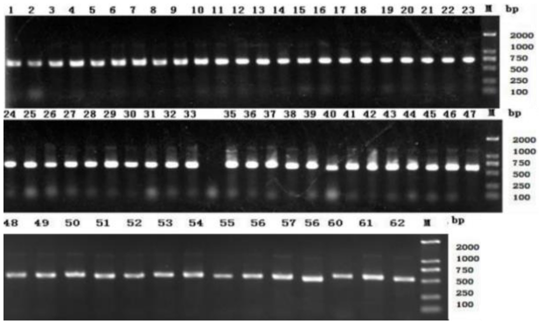

Fig.S2 The electrophoresis profile of tef1-α of the isolates.

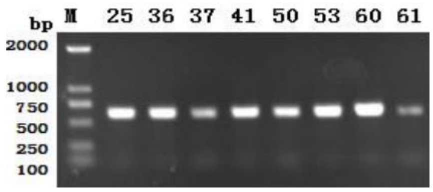

Fig.S3 Effect of different temperature treatment of mycelia growth of 2 *L. edodes* and *Trichoderma* spp. isolates.

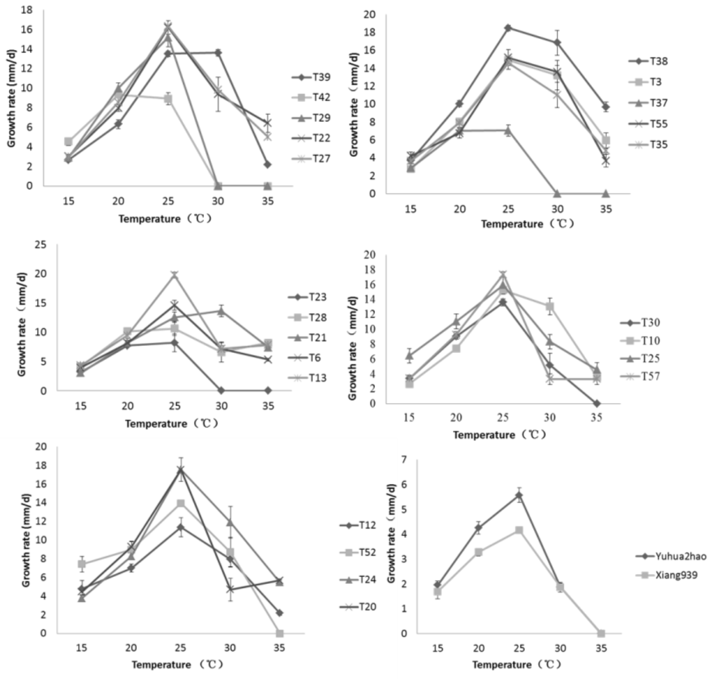

Note: *T. harzianum*: T3, T6, T10, T12, T20, T21, T27, T28, T38, T42, T55; *T. atroviride*: T25, T29, T30, T24; *T.*

viride: T13, T23, T52; *T. pleuroticola*: T22, T35, T39; *T. longibrachiatum*: T57; *T. oblongisporum*: T37.

Fig.S4 Effect of different pH treatment on mycelia growth of two *Trichoderma* isolates and one *L. edodes* strain.

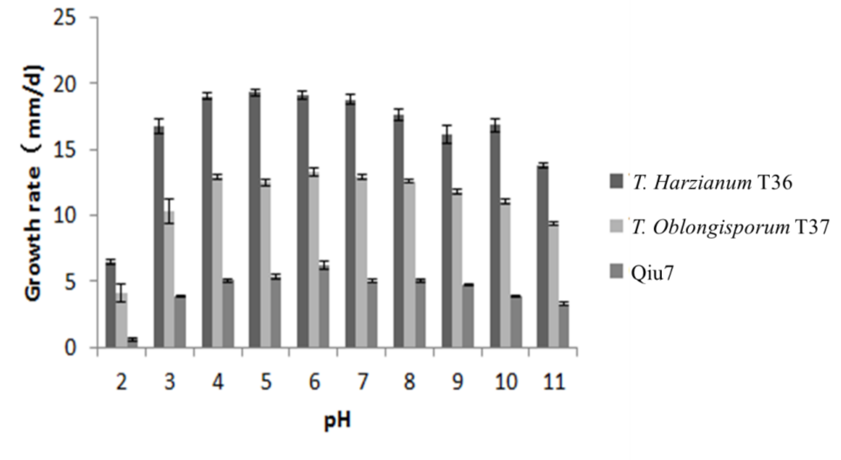

Fig.S5 Inhibition rate of 6 *Trichoderma* species on *L. edodes* Qiu-7 in confrontation culture.

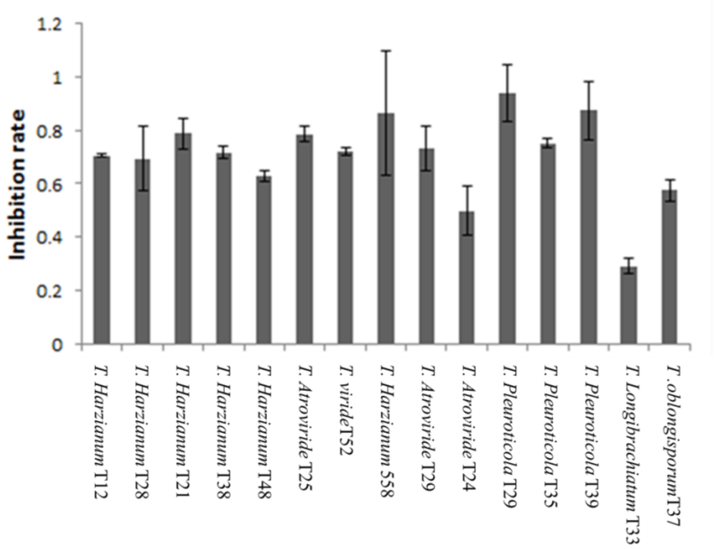

Supplement: Supplementary file 1 — Figure S1. The electrophoresis profile of ITS of the isolates. Figure S2. The electrophoresis of tef1‐α of the isolates. Figure S3. Effect of different temperature treatment of mycelia growth of 2 L. edodes and Trichoderma spp. isolates. Figure S4. Effect of different pH treatment on mycelia growth of two Trichodermaisolates and one L. edodes strain. Figure S5. Inhibition rate of 6 Trichodermaspecies on L. edodes Qiu‐7 in confrontation culture. [file MBO3-5-709-s001.pdf]
